# Identification of extracellular vesicles from their Raman spectra via self-supervised learning

**DOI:** 10.1038/s41598-024-56788-7

**Published:** 2024-03-21

**Authors:** Mathias N. Jensen, Eduarda M. Guerreiro, Agustin Enciso-Martinez, Sergei G. Kruglik, Cees Otto, Omri Snir, Benjamin Ricaud, Olav Gaute Hellesø

**Affiliations:** 1 Department of Physics and Technology, UiT The Arctic University of Norway, Tromsø, Norway; 2 Thrombosis Research Group (TREC), Department of Clinical Medicine, UiT The Arctic University of Norway, Tromsø, Norway; 3 Oncode Institute and Ten Dijke/Chemical Signaling Laboratory, Department of Cell and Chemical Biology, Leiden University Medical Center, Leiden, The Netherlands; 4 Amsterdam Vesicle Center, Department of Biomedical Engineering and Physics, Amsterdam University Medical Centers, Amsterdam, The Netherlands; 5 Laboratory of Experimental Clinical Chemistry, Department of Clinical Chemistry, Amsterdam University Medical Centers, Amsterdam, The Netherlands; 6 CNRS, Institut de Biologie Paris-Seine, Laboratoire Jean Perrin, Sorbonne University, Paris, France; 7 Department of Medical Cell BioPhysics, TechMed Centre, University of Twente, Enschede, The Netherlands; 8 Department of Medical Biology, UiT The Arctic University of Norway, Tromsø, Norway

**Keywords:** Raman spectroscopy, Cellular microbiology, Scientific data, Nanoscale biophysics, Endosomes

## Abstract

Extracellular vesicles (EVs) released from cells attract interest for their possible role in health and diseases. The detection and characterization of EVs is challenging due to the lack of specialized methodologies. Raman spectroscopy, however, has been suggested as a novel approach for biochemical analysis of EVs. To extract information from the spectra, a novel deep learning architecture is explored as a versatile variant of autoencoders. The proposed architecture considers the frequency range separately from the intensity of the spectra. This enables the model to adapt to the frequency range, rather than requiring that all spectra be pre-processed to the same frequency range as it was trained on. It is demonstrated that the proposed architecture accepts Raman spectra of EVs and lipoproteins from 13 biological sources and from two laboratories. High reconstruction accuracy is maintained despite large variances in frequency range and noise level. It is also shown that the architecture is able to cluster the biological nanoparticles by their Raman spectra and differentiate them by their origin without pre-processing of the spectra or supervision during learning. The model performs label-free differentiation, including separating EVs from activated vs. non-activated blood platelets and EVs/lipoproteins from prostate cancer patients versus non-cancer controls. The differentiation is evaluated by creating a neural network classifier that observes the features extracted by the model to classify the spectra according to their sample origin. The classification reveals a test sensitivity of $92.2\%$ and selectivity of $92.3\%$ over 769 measurements from two labs that have different measurement configurations.

## Introduction

Extracellular vesicles (EVs) are nanostructures confined by a lipid bilayer, produced by all types of cells, and released into the extracellular space. EVs play crucial roles in cell-to-cell communication and coagulation. Given their abundance in biological fluids and circulation, they have been identified as biomarkers for various inflammatory diseases and cancer^1–4^. This has increased the interest in EVs and the development in the field, as documented in recent reviews^5,6^. The biochemical composition of extracellular vesicles (EVs) is highly heterogeneous. The nanoscale size of EVs and other biological nanoparticles poses a significant challenge regarding analysis, and the field still lacks standardized analytical approaches. Currently, researchers employ several methods, including nanoparticle tracking analysis (NTA)^7^, transmission electron microscopy (TEM), flow cytometry^8^, and various chemical/biological techniques^9^. NTA measures the size distribution of EVs by tracking their Brownian motion. While it provides valuable size information, it does not offer insights into the biochemical properties of EVs, and size distribution alone is not sufficient to attribute EVs to their cellular origin. To use EVs as a biomarker for various diseases, suitable characterization and data analysis methods must be found.

Raman spectroscopy is a label-free methodology providing information about chemical composition. It is a viable alternative to established chemical analysis tools, such as magnetic resonance and mass spectrometry^10,11^. By combining Raman spectroscopy with optical trapping, single particles down to nanoscopic sizes can be captured and measured independently^12,13^. This combination is very promising for characterization of EVs. However, it is a challenge to decode the Raman spectra into the corresponding chemical mixture. For simple materials, such as polymers, decomposing the Raman spectra is relatively easy due to the inherent sparsity of vibrational modes in such materials^14,15^. For more complex samples, notably biological materials, their features are both numerous and often overlapping^16–18^. As an example, the true proportion of each biomolecule in EVs is rarely available, and there is thus no benchmark for decoding the Raman spectra. Furthermore, as Raman scattering is very weak, the spectra contain various amounts of noise (on y-axis) and the wavelenth shift (along x-axis) can be stretched or shifted, depending on the optical spectrometer and its calibration. To further complicate the picture, different labs have different set-ups, giving Raman spectra with different range for the wavelength shift.

Our aim is to make a flexible model that can extract high-quality, chemically significant information from Raman spectra and use this information to classify EVs and other biological nanoparticles. The model should be able to handle noise and variations in wavelength calibration, which typically occur when using data from multiple sources. This complex challenge requires a flexible analysis method that can handle large variations in the input, but still extract information that reflects the chemical composition of the EVs and other nanoparticles.

Common approaches to analyse Raman spectra are signal processing and analysis methods that decompose the spectra into their more fundamental components, which can, in some cases, be associated with known chemicals. Principal component analysis^19^ and k-means clustering^20^ are two common methods applied for this purpose. They are often complemented by a classification method, such as linear discriminant analysis^21^ or a support vector machine^22^. A method has also been proposed for relating Raman spectra to the biomolecular composition of the sample, called biomolecular component analysis^23^. Some of the data presented here (from Sorbonne University) has been analysed by biomolecular component analysis^24^. While these methods have demonstrated their usefulness, they are limited by their relatively simple function and thus their limited ability to consider complex patterns and dependencies in data. Neural networks and deep learning are very efficient methods for analysing data. Their ability to model highly non-linear relationships allows them to perceive complex patterns in the data. This has made neural networks, and derivatives such as convolutional neural networks, prevalent in the field of data analysis and they have been successfully applied on spectral information^1,25,26^. Another significant advantage of neural networks is their adaptability to noise in the data and variations in the signal background^1,27^. However, these methods often require several thousand examples to learn from and these examples have to be rigorously curated to avoid biasing the model. The last requirement often implies that the setting of the data must be made uniform, with the same frequency range and resolution for all spectra. This puts strict restrictions on the data that can be used, and calibration drift is a common experimental problem which is not acceptable to such a model. For data from multiple sources with differences in range and resolution, the solution becomes pre-processing of the data by truncation, interpolation and normalization, in an attempt to emulate uniform settings.

We propose a neural network architecture specially tailored to handle Raman spectra from samples for which we only have a few labels. It is based on a self-supervised training approach. The architecture and training take care of the specificities of the spectra, of the noise properties from the measurements and on the variability of the recording from different instruments and labs. The general achitecture is based on a Variational AutoEncoder ^28^ (VAE). However, since the data from multiple instruments can have different ranges, the autoencoder uses a novel formatting. It considers the signal (y-axis) and the frequency range (x-axis) separately in order to handle both noise (on y-axis) and changes in wavelength calibration (x-axis). This split is also taken advantage of to equalize the input data size by applying a resampling step with interpolation. The architecture further includes a suitably sized latent space and a loss function adapted to picking-up the significant spectral information.

We adopt the standard approach in self-supervised learning ^29^. First, we train the autoencoder in a self-supervised manner, i.e. without labels. Training data are generated by adding Gaussian noise, wavelength shift and clipping of the original spectra. In addition, the training data contains spectra of both EVs and lipoproteins, and is augmented with pure noise spectra, emulating the case of no trapped particle. The task of the network is to recover the original spectra from the artificially corrupted ones. While training on this data, the network will identify which information in the input is important in order to recover the original spectra ^30^. The network will build an inner representation of the data in the latent space. Data encoded in the latent space should contain only the essential information to reconstruct the original spectra, without any noise, and this information will ideally approximate the biochemical information of the particles.

In the second stage of the learning process, we take advantage of the latent space and use it as the input of a second, deep neural network which will learn to associate the data to labels given by the origin of the EVs. It is an advantage to use the latent representation for classification as it should reflect the biochemical information of the particles and be free from noise. The classification task is thus made easier, allowing the use of a smaller network and a reduced need for labelled data. Further details are given in the next section.

Raman spectra from two laboratories, at University of Twente (Netherlands) and at Sorbonne University (France), are used to train and test the model. For simplicity, the two datasets are referred to as ‘Twente’ and ‘Paris’. The Twente dataset was larger than the Paris dataset, which gives an imbalance when training the model. Variations in measurement method, notably excitation wavelength and acquired wavelength range, and in sample preparation can give features and distortions in the data that the model should learn to disregard, thus recognizing the particles more by their biochemical information rather than the conditions of the data. For one case, the two laboratories analysed EVs and lipoproteins derived from the same cell type: blood platelets. For this case, it is important to see if the model recognizes the origin of the particles or at which lab they were analysed.

The performance of the model regarding reconstruction of spectra, extraction of information-rich features, and classification is evaluated. The classification of EVs based on the information extracted by the model is used to verify the quality of the information and the viability of using Raman spectra of EVs as biomarkers for a range of conditions. The analysis methodology proposed in this article will be a valuable tool in that process, as it can handle data from several laboratories with variations in measurement settings. These problems and differences between datasets can be found for many applications, including other types of spectroscopy and in general for one-dimensional datasets from different sources.

## Methods

### Samples

The samples considered in this work are EVs and lipoproteins from 13 biological origins, acquired with two different measurement systems in two different laboratories. Out of the 13 origins, three are commercially available cell line cultures, two are from bulk human blood, two are from platelets, two are derived from donor plasma , and the last four are pure lipoproteins. See supplementary information and Table S1 and S2 for an overview.

The three cell lines used are LNCaP, PC3 and THP-1. It is investigated if they are recognized as different from the human-derived samples and how they are grouped relative to each other. The two samples from bulk blood are EVs taken from blood plasma and red blood cells (hence RBC) to investigate how the model perceives their similarity and difference. The two samples from platelets are isolated from the other elements of blood and are presumed to be from pure cultures. These are included to investigate whether Raman spectra of their EVs and/or lipoproteins can be used to differentiate resting platelets from activated platelets, which contribute to blood clotting^31^. The data from platelet-derived EVs/lipoproteins come from four sets:

1. Control: platelet EVs from untreated platelets from donors prepared at the University of Tromsø and measured at the Sorbonne Université
2. Activated A23187: EVs from artificially activated platelets using Calcium Ionophore (A23187) prepared at the University of Tromsø and measured at the Sorbonne Université
3. Activated TRAP6: EVs from artificially activated platelets using a Thrombin-activator (TRAP6) prepared at the University of Tromsø and measured at the Sorbonne Université
4. Mixed: mix of EVs and lipoproteins derived from platelet concentrate prepared by and measured at the University of Twente

The two datasets from plasma-derived samples originate from five patients afflicted with prostate cancer and five from non-afflicted controls. These datasets are included to investigate if the model can determine if the EVs and/or lipoproteins originate from a healthy or an afflicted person and to see how these two are clustered relative to each other. Lastly, the four types of lipoproteins (CM, HDL, LDL and VLDL) are included to investigate how the model reacts to a mix of lipoproteins and EVs. This also poses a special challenge for the model as the number of spectra from lipoproteins is comparatively low and, due to their small size, also has a very low signal-to-noise ratio. See supplementary information and Tables S1 for more information about the samples and sample preparation. Regarding the ‘Paris’ dataset, collection of clinical samples has been approved by the regional ethical committee for Medical and Health Research Ethics (REK80025). All participants were above the age of 18, did not suffer from illness, or use medication; all gave a written informed consent. Regarding the ‘Twente’ dataset, blood was obtained from non-fasting healthy donors and cancer patients (both N=5) after written informed consent in accordance with the Helsinki Declaration and approved by the medical-ethical assessment committee of the Academic Medical Center, University of Amsterdam (NL 64623.018.18).

### Data acquisition

The Raman spectra of the various EVs and lipoproteins were collected with optical tweezers using two separate measurement systems^12,32^. In both systems, the trapping and excitation of the Raman scattering is performed by a single, high-power laser relayed by a high-NA objective. The same objective also collects the Raman scattered light in backpropagation mode.

In system 1, built by Dr. Sergei G. Kruglik at Sorbonne University, the laser source is a Ti:Sapphire laser (Model 3900S, Spectra Physics, USA), pumped by a DPSS laser (Millennia eV, Spectra Physics, USA) delivering approximately 180 mW at 780 nm to the sample volume via a water dipping objective (Olympus LUMFL, 60X, NA = 1.1). The backpropagated response is collected by the objective and passed to a 500mm focal length grating spectrograph with a liquid nitrogen cooled detector and a 50$\mu m$ slit aperture, acquiring the spectra over a range of 309–2035 cm^-1^. The sample particles are suspended in a solution of PBS which is in direct contact with the objective lens. The high numerical aperture and diluted concentration of particles in the suspension allows the system to trap single or few (<5) particles at a time. Spectra are then acquired from the trapped particles using an integration time of 3 seconds per frame and averaged over 30–50 frames to generate a spectrum of the trapped particle(s).

In system 2, built by Ing. Aufried Lenferink at University of Twente, the laser source is a Krypton-ion laser (Coherent, INNOVA 90-K) delivering 70 mW at 647 nm to the sample volume via a non-immersion objective (Olympus, 40x, NA = 0.95). The backpropagated response is collected by the objective and passed to a prism-based spectrometer built in-house with a Peltier-cooled detector, acquiring the spectra over a range of 301–3655 cm^-1^. The sample particles are suspended in PBS in an enclosed volume using a coverslip to prevent interaction with the environment. Using an objective with relatively high numerical aperture and monitoring of the Rayleigh scattering intensity of the spectra, single particles are trapped and measured^32^. Each spectrum in the Twente set thus corresponds to a single particle, measured with an integration time of 38 ms per frame and typically averaged over 256 frames to generate the spectrum of the single trapped particle.

### Datasets

**Figure 1:**
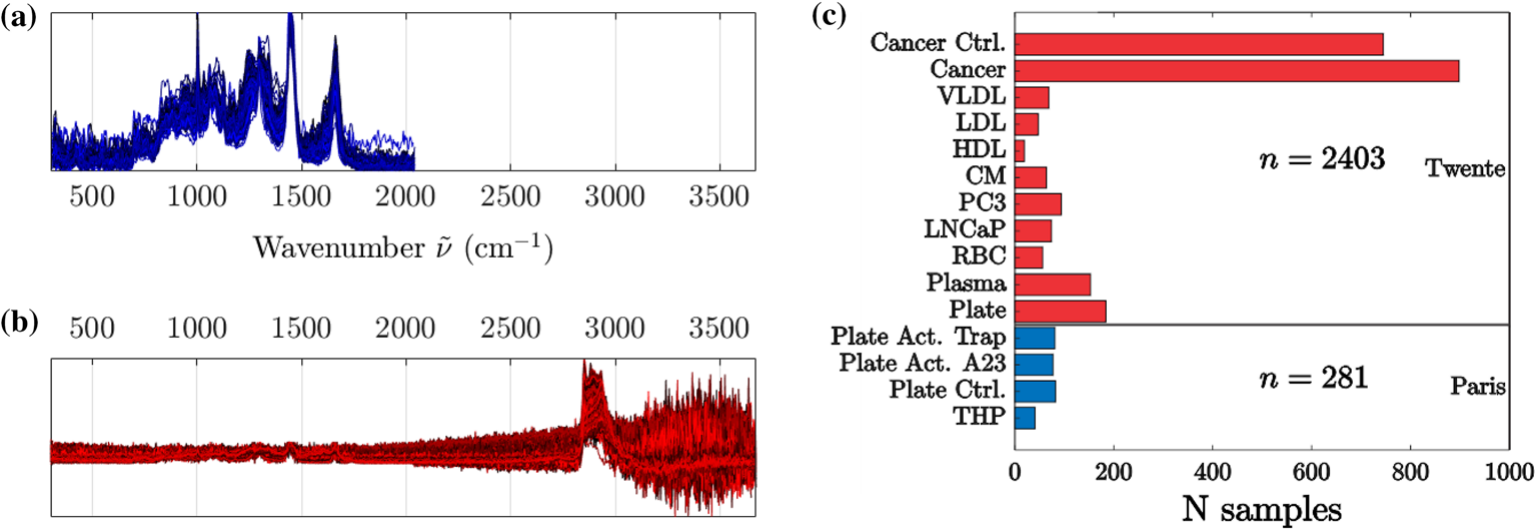
The spectra making up the entire dataset used in this work. The spectra from Paris, shown in (**a**), have a wavenumber range of 307–2041 cm^-1^, while the spectra from Twente, shown in (**b**), have a wavenumber range of 300–3674 cm^-1^. The distribution of spectra to each biological origin is shown in (**c**), with 2403 spectra from Twente and 281 spectra from Paris.

The data used in this work consists of 2684 spectra from 13 origins as described previously and listed in Table S2. One of the challenges is that the wavenumber range and center varies from spectrum to spectrum, especially since the ‘Paris’ dataset originates from a system with a much shorter wavenumber range than the ‘Twente’ dataset. The difference in the wavenumber range is shown in Fig. 1a,b, and the figure also shows the difference in the signal-to-noise ratio between the two datasets. There is also a significant variation in the preparation of the samples, particularly the isolation of the particles, as the protocols and concentrations vary between the two labs. Figure 1c illustrates the number of spectra from each biological sample. The mean Raman spectra for each or the origins are shown in the supplementary material (Fig. S1) with chemically significant spectral bands highlighted. The expression of these features for each of the biological origins is also shown, with mean and standard deviation (Fig. S2). There is a difference between the spectra from each biological origin but also a significant standard deviation, making classification based solely on these spectral features challenging. Other works characterizing some of the particles in the Twente dataset using select bands and PCA have also shown interesting differences between the spectra^33^.

### Code availability

The code for the developed autoencoder architecture (see next section) is available at: github.com/MJE059/Raman-convolutional-autoencoder.

**Figure 2:**
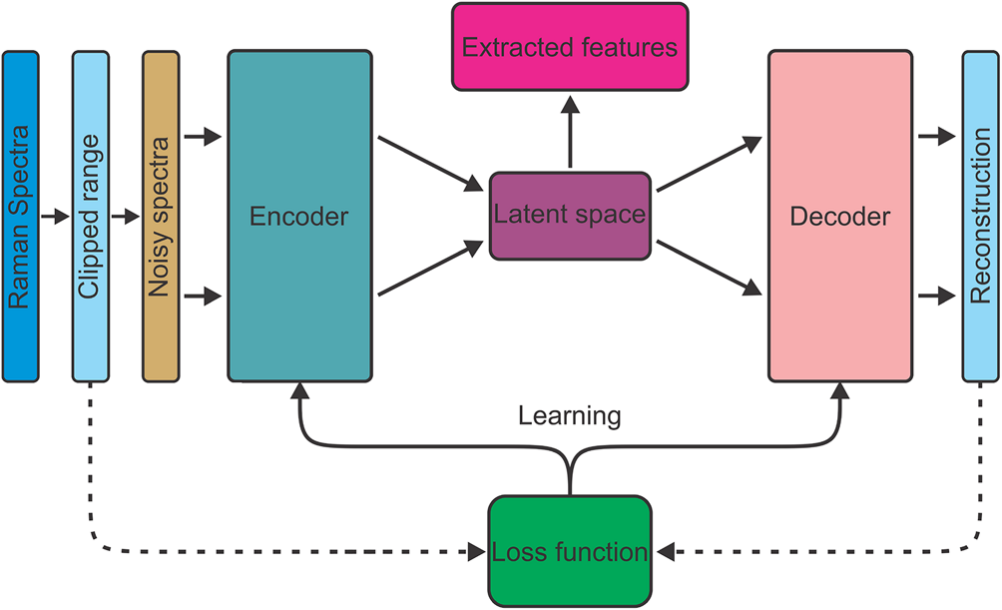
Schematic of the autoencoder. The encoder takes the vector of noisy and/or clipped Raman spectra as input and compresses it into the latent space, which contains the extracted features. The decoder takes the latent space as input and attempts to reconstruct the original Raman spectra. Learning occurs by computing a loss between the original Raman spectra and the reconstructed spectra and passing the gradient to the encoder and decoder.

## Machine learning methodology

**Figure 3:**
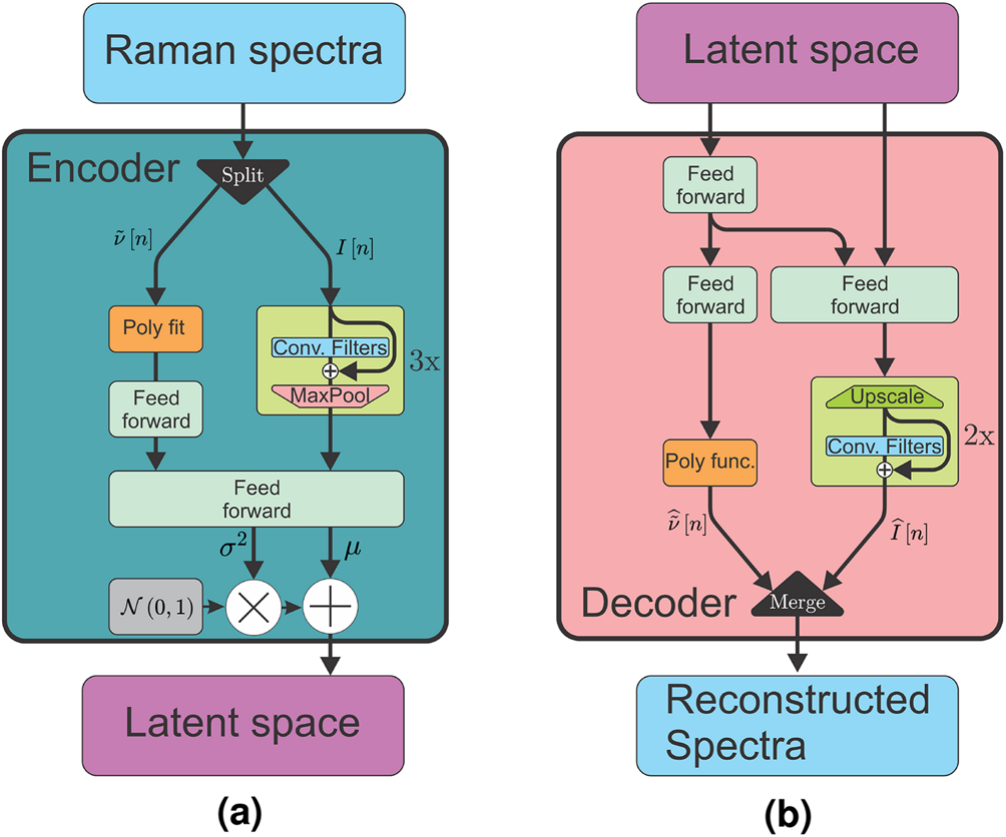
Schematic of the encoder (**a**) and decoder (**b**) of the autoencoder architecture. The encoder splits the input into an intensity vector $I\left[ n \right]$ and a wavenumber vector $\tilde{\nu }\left[ n \right]$, which follows two paths. The two paths with frequency (x) and intensity (y) only meet in the main feed forward network and in the latent space. Similarly for the decoder in (**b**), the latent space data is split to follow two paths. The frequency vector is reconstructed using a polynomial whose parameters are given by feed forward layers and the intensity vector is reconstructed by convolutional filters.

The desired output from the self-supervised, deep neural network is chemically relevant information extracted from the Raman spectra, with sufficient quality to reliably group particles according to their biochemical nature. To achieve this, we apply an autoencoder^28,34^ to the Raman spectra, as shown in Figs. 2 and 3. The goal of the encoder is to learn to extract the most valuable information from the spectra and pass this on to the latent space. The quality of this information is verified by the decoder, whose purpose is to reconstruct the original spectra using only the information available in the latent space. If the quality and completeness of this information is high, the decoder can reconstruct the spectra with high accuracy.

In order to extract the important information, spectra are first clipped at random positions, removing the beginning and/or end. This forces the network to be robust to inputs with different spectral ranges. Second, noise of different amplitude is added to the clipped spectra. The loop for the self-supervised phase 1 learning is closed by computing a loss, which quantifies the amount of information lost between the original (possibly clipped) spectra and the reconstructions produced by the decoder. During this phase, the autoencoder learns to reject noise and becomes robust to clipping; whatever spectra is passed to the encoder should produce an approximately equivalent representation in the latent space, containing high quality information that reflects the biochemistry of the sample. Phase 2 learning commences by interfacing the classifier with the latent space of the autoencoder and passing the training data through the encoder such that a training set is generated in the latent space. The classifier relies on this training set and labels with the known origins of the EVs for its learning.

### Variational autoencoder

The Raman spectra are naturally continuous along the frequency axis, and therefore we base our autoencoder architecture on convolutional layers. To facilitate better learning of a deep model, we also implement skip connections across the convolutional layers, akin to those introduced in the ResNet architecture^35^. This allows the gradient to bypass the layers during learning. We also implement Gaussian re-sampling as is used in variational autoencoders^36^ to encourage the learning of uncorrelated features in the latent space.

The autoencoder should learn features in the Raman spectra that correspond to biochemical aspects in the sample, making it preferable for one such biochemical aspect to be represented in a single dimension of the latent space. By using a variational autoencoder, which attempts to learn the latent features as uncorrelated gaussian variables, the model is motivated to express the features as independently as possible. Our hypothesis is that this motivates the network to express patterns that correlate strongly, such as the $CH_2$ peaks and lipid complexes, in one latent space dimension and other spectral signatures that correlate weakly, such as the amino and carotenoid complexes, into separate dimensions. Thus, the learned representations of a single biochemical should be expressed in a single latent dimension instead of being spread out over multiple dimensions. That way, we hypothesise that the latent space will form a more realistic, and thus more valuable, map of the biochemical features in the sample, making separation of the different classes of EVs more easy.

### Adaptive frequency range

A variational autoencoder based on convolution can process spectra expressed as intensities only, but this would deprieve the model of essential information about the frequency range. When interpreting the Raman spectra of a sample, be it by a human or by a machine, identifying both the peak intensity and its position in the spectrum is crucial. Conventionally, this information is passed to the model *implicitly* by pre-processing the dataset such that each index of the spectrum vector corresponds to the same wavenumber shift. The model thus learns to associate a peak with an underlying component (i.e. a chemical) by where the peak appears in the vector rather than its wavenumber shift. In our context, this is not possible as the spectra have different ranges. Instead, we propose making information regarding the frequency range available to the model such that it can be considered *explicitly*. This is implemented in the encoder as shown in Fig. 3a by passing the intensity and the frequency vectors to the model on two separate channels, and allowing them to follow two information pipelines through the model. The intensity vector passes through convolutional filters that extract the spectral features, while a fourth degree polynomial is fitted to the frequency vector. The fitted parameters are first pre-processed by a feed forward block, before being concatenated with the spectral feature information. Frequency and feature information are processed together in the main feed-forward block of the encoder. Similarly, as shown in Fig. 3b, the decoder extracts the frequency related information from the last ten dimensions of the latent space, processes it through two feed forward blocks to find the polynomial parameters, and reconstructs the frequency range using the parameters and a fourth degree polynomial. The intensity vector is reconstructed by passing the frequency and intensity related information from the latent space to a feed forward block whose output goes through a series of up-sampling convolution filters. The reconstructed frequency and intensity vectors are finally concatenated to produce an output of the same format as the input. The final architecture has a total depth of 36 layers including the adaptive frequency neurons, making the model quite deep. Further details on the architecture are available in Table S3.

#### Loss function

During training, the autoencoder should learn to reconstruct the data as accurately as possible. Phase 1 learning must use a loss function whose metrics reflect this and motivates the learning to extract biochemical information from the spectra. For this, it was found advantageous to add Fourier loss to the more standard spatial and Kullback-Leibler losses. *The spatial loss function* describes the difference between the clipped original spectra and the reconstruction in the most direct manner and serves as the most fundamental loss function for the autoencoder during phase 1 learning. The metric of this loss is the root mean square error between the original and the reconstruction. *The Kullback-Leibler loss function* describes the difference between the discrete distributions in the latent space and a gaussian distribution. The need for this loss arises from the gaussian re-sampling in the last layer of the encoder and is described by the Kullback-Leibler divergence between the output of the last feed forward layer in the encoder, which produces the means $\mu$ and variances $\sigma ^2$ for the re-sampling, and a a gaussian distribution with a mean of zero and a variance of 1. Thus, the more the latent representations approximate uncorrelated gaussian distributed variables, the lower the KL loss will become. *The Fourier loss function* describes the difference between the original and the reconstruction in Fourier space. The metric of this loss function is the sum of the mean square difference of the frequency and phase between the input and the reconstruction. This loss is included to counter unwanted effects of the simple spatial loss function. The spatial loss is most sensitive to low-frequency features ^37^ such as large, smooth slopes. This encourages the autoencoder to consider the low-frequency elements more strongly, thus implicitly learning to low-pass filter the input ^38^. By adding a Fourier loss, the autoencoder is forced to consider the higher frequency elements as well and, by adding a mask that attenuates intermediate frequencies, it is encouraged to preserve high-frequency elements of the spectra, such as sharp peaks.

*The total loss function* is a composite loss formed by these losses:

$$\begin{aligned} L_\Sigma = (1-\alpha )\cdot (L_{RMS,I}+\gamma L_{RMS,\tilde{\nu }}+\beta L_{KL}) + \alpha L_{Fourier}, \end{aligned}$$

where $L_{RMS,I}$ is the RMS difference between the original and reconstructed intensity vectors, $L_{RMS,\tilde{\nu }}$ is the RMS difference between the original and reconstructed frequency vectors, $L_{KL}$ is the Kullback-Leibler loss, and $L_{Fourier}$ is the Fourier loss. The balancing parameters $\alpha$, $\beta$ and $\gamma$ are used to moderate the relative strength of the losses. The parameter $\alpha$ balances the ratio of the Fourier loss relative to the other losses, and it is set to 0.3 during training to make the spatial and KL losses slightly dominant over the Fourier loss. The parameter $\beta$ determines the strength of the KL loss and is set to 5 to ensure the KL loss is significant during learning, but not clearly dominant. The parameter $\gamma$ balances the loss of the frequency reconstruction versus the intensity reconstruction to compensate for the different magnitudes of the two vectors. In training, the $\gamma$ parameter is set to $10^2$ to compensate for the frequency vector having a magnitude in the lower range of $10^3$ while the intensity vector can have magnitudes approaching $10^5$.

### Classifier head

**Figure 4:**
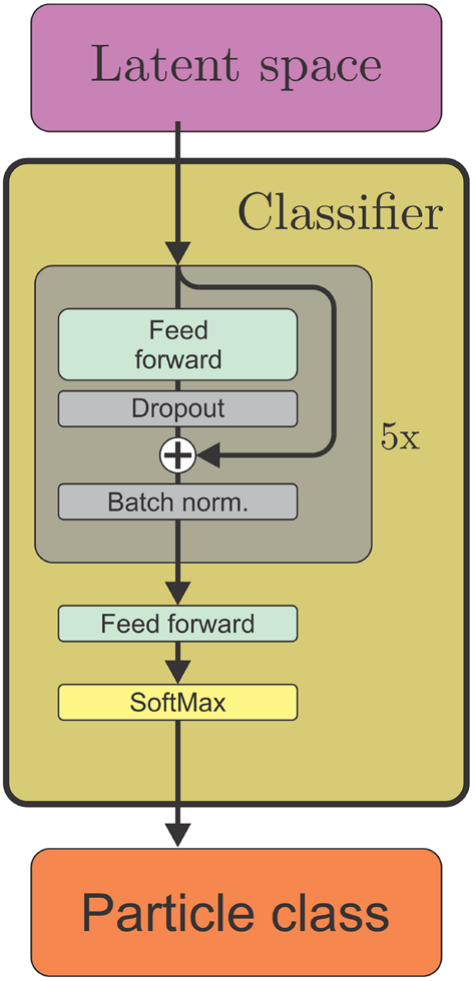
Schematic of the classifier head. The latent space representations of the spectra are passed to five blocks of fully connected feed forward layers with dropout and optional skip connections. The output of the last block is passed to a final feed forward layer with softmax activation which yields the predicted particle class.

The classifier head, shown in Fig. 4, is separated from the autoencoder and only passively interacts with it through observing the latent space. It performs classification of the spectra through a conventional feed forward-approach where the latent space is passed to a series of feed forward networks with dropout, batch normalization, and skip connections. Due to the high level of pre-processing by the encoder, this network can be made relatively small, consisting of five layers of 128 neurons only. The output of this processing is passed to a classification head consisting of a feed forward layer with a softmax activation, which produces a one-hot encoded class prediction.

### Training scheme

#### Artificial noise in training

As shown in Fig. 1, the available spectra forms two distinct datasets, one from Paris and one from Twente. The most distinct differences between these two datasets are in signal-to-noise ratio, in frequency range, and in calibration drift. The autoencoder must be trained to be robust against these differences. To achieve this, the training set was augmented with three types of noise : noise in the intensity to emulate variations in the signal-to-noise ratio, clipping of the frequency range to emulate different acquisition ranges, and distortions in the frequency axis to emulate calibration drift. *The intensity noise* is implemented as additative Gaussian noise in the intensity of the spectra. In training, the variance is set to $-5$ dB of the RMS of the intensity before noise, calculated on a per-spectrum basis to make the significance of the noise similar between the spectra. *The range clipping* is implemented by selecting a random start and stop for the range and removing the parts of the spectrum that is outside this range. The start is kept below a prescribed maximum, set to 800 cm^-1^, and the stop is kapt above a minimum, set to 1500 cm^-1^. This is decided such that the fingerprint-region (800–1500 cm^-1^) is always kept while parts of the spectra outside this is randomly removed. This results in up to 79 $\%$ of the spectrum being clipped. *The frequency noise* is implemented as a distortion in the frequency vector. As previously described, a fourth degree polynomial is fitted to the frequency vector: $P_4[I,;\beta ]=\beta _4I^4+\beta _3I^3+\beta _2I^2+\beta _1I+\beta _0\approx \tilde{\nu }[I]$. The parameters $\beta _n$ emulates the parameters determined when calibrating the spectrometer, poor calibration is emulated by adding distortion to each of the parameters $\beta _n$. The amount of distortion $\hat{\beta }_n$ is set to 2$\%$ of $\beta _n$, giving a distorting polynomial $\hat{P} [I,\hat{\beta }]$. This distorting polynomial is added to the frequency vector to emulate poor calibration of the spectrometer: $\tilde{\nu }_N [I] = \tilde{\nu }[I] + \hat{P} [I,\hat{\beta }]$.

A last addendum to the training set is a set of randomly selected spectra whose intensities are purely Gaussian noise. These are included to force the model to recognize a zero-signal condition, and thus discourage it from considering the spectra as variations on a “mean” spectrum. The intention is to encourage the model to consider the spectra as intrinsically unique, and also express them as unique in the latent space. Experiments have shown that this is beneficial (not reported here).

#### Training phases

The raw spectra are first separated into the training set (70$\%$, 1898 spectra) and the testing set (30$\%$, 769 spectra) which are kept isolated from each other in the data structure. During phase 1 training, a new noisy set is generated from the raw training set for each epoch of training to encourage the model to be robust against noise. These training sets are generated by adding together one copy of the raw training set, three copies with the described noise types, and one copy of noise-only signals. The generated training set is then randomly shuffled before being passed to the model for training. The model is then built with the architecture illustrated in Fig. 3 and described in Table S3 in the supplementary information. The built model is allowed to train on the generated training sets for 100 epochs with a learning rate of $10^{-4}$ before concluding phase 1 of the learning. Once trained, the encoder of the model is used to generate latent representations of the training data which is then used to train the classifier. The classifier is fed the latent training set and tasked with classifying the data into 13 classes corresponding to the known particle origins which are used as labels. To compensate for the variation in the number of spectra of each class, as shown in Fig. 1c, a soft balancing scheme is used to weight the loss of the classification during training. The class weighting is calculated from the distribution of the classes in the dataset and takes the form of a sigmoid function. As the representation of a given class in the set approaches 100$\%$ of the number of spectra in the set, the weight of the class approaches 0.1. Conversely, if the representation of a class approaches 0$\%$ of the number of spectra in the set, the weight of the class approaches 1.

**Figure 5:**
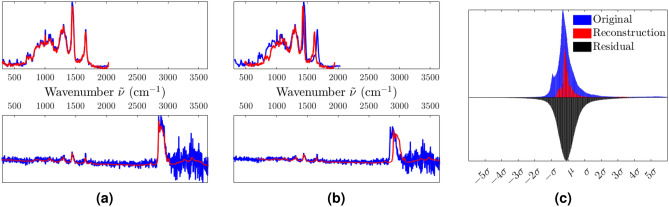
Reconstruction performance of the model, showing the input in blue and the reconstruction in red. The model is tasked with reconstructing the same data, but only the intensity vector in (**a**) and both frequency and intensity vectors in (**b**). The histograms in (**c**) show the statistical distribution of the original spectra, of the reconstructed spectra, and of their residual difference.

## Results

### Reconstruction performance

The first measure of the quality of the model is the accuracy of the reconstructions it produces. The trained network is provided with unaltered spectra from the test set and asked to reconstruct them, producing the results shown in Fig. 5. Note the significant reduction in noise in both cases and the preservation of sharp features, such as the phenylalanin peak at 1003.6 cm^-1^. Figure 5b shows that the reconstruction of the frequency vector is not perfect, producing an elongation or compression of the spectra. However, the reconstruction is adapting to changes in the range, approximates it well, and, most importantly, the shapes of the features are preserved. By comparison, truncating and interpolating the data removes a significant part of the spectra, see Fig. S3 in the supplementary material.

The most notable difference between the original spectra and the reconstructions is the significant noise reduction, especially for high wavenumbers (> 3000 cm^-1^). The autoencoder is trained on data with artificial noise and has learned to remove this noise. Therefore, when presented with spectra from the test set that only have natural noise, it will attempt to remove the natural noise in the same way. This process can be evaluated by investigating the difference between the original and the reconstructed spectra. The distribution of this residual is shown in Fig. 5c. In the case of perfect noise removal, the residual should consist of noise, which is presumed to have a Gaussian distribution. The Kullback–Leibler divergence between the distribution of the residual and a Gaussian distribution reveals a normalized divergence 3.3 times lower than for the original spectra, indicating that the residual is largely Gaussian and thus mostly noise.

### Feature extraction capabilities

The high reconstruction accuracy of the model indicates that the information preserved in the latent space is of high quality and that noise is not preserved. The next step is to evaluate the relevance and significance of the information as a means to characterize the sample. This is done by passing the test data through the encoder of the network and extracting the predicted means of the latent space distributions such that the “perception” of the encoder can be investigated.

**Figure 6:**
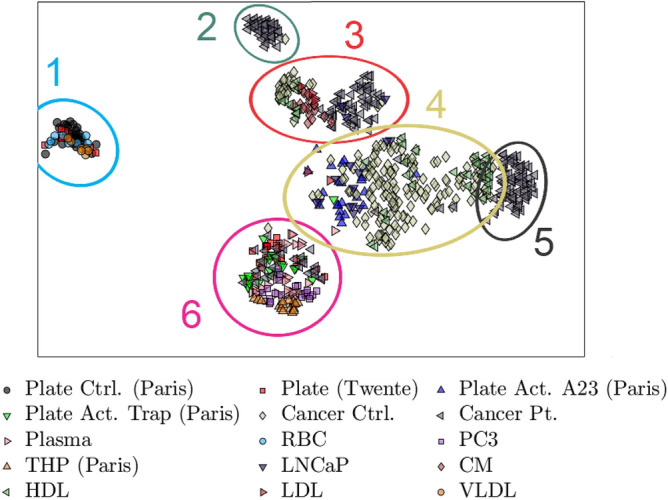
Two-dimensional t-SNE projection of the latent representations of spectra from the test set, giving a general overview of the latent space. Six clusters can be seen, showing that the network is able to “see” similarities in the data. Cluster 1 contains the control platelets from Paris with some from Twente, the red blood cells, the VLDL lipoproteins and some of the cancer control particles. Cluster 2 contains some of the particles derived from cancer patients with slight overlap with those derived from cancer controls. Cluster 3 contains particles from both the cancer and cancer control particles, as well as the LDL lipoproteins, Chylomicrons, and some of the HDL lipoproteins. Cluster 4 contains the bulk of the cancer control particles and all of the A23187-activated platelets (A23). It also contains the remaining HDL lipoproteins, the LNCaP-derived particles and the cluster overlaps with the plasma particles. Cluster 5 contains almost solely cancer patient derived particles. Cluster 6 is a mixed cluster containing the PC3- and THP-derived particles, the TRAP6-activated platelets (Trap), and the bulk of the plasma and platelet particles from Twente.

By using t-distributed stochastic neighbour embedding (t-SNE) ^39^, we project the 100 dimensional data in the latent space down to two dimensions and use the known origins of the data as labels to illustrate the latent space representations as shown in Fig. 6. The t-SNE projection is useful to show groupings in the latent space, but because of the compression from 100 dimensions to two dimensions, the projection will not be a true representation of the latent space. The principle of t-SNE is to reduce the dimensionality while keeping points that are close in the high dimensional space close in the low dimensional one. Points in a t-SNE plot do not reflect where they are in the latent space, but their proximity to other points reflects their proximity in the latent space. Note that points in the t-SNE plot may appear more clustered than they are in reality ^40^. Hence the clusters in the next figures indicate that points are located close to each other in the latent space but they may not be well isolated from the others.

**Figure 7:**
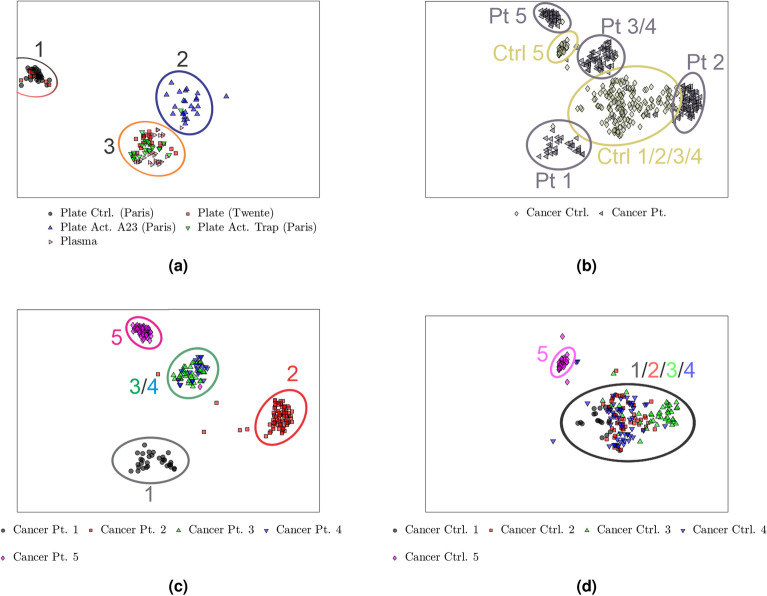
Two-dimensional t-SNE projection of latent space EVs and particles derived from (**a**) platelets and plasma, (**b**) prostate cancer patients and controls, (**c**) prostate cancer patients, and (**d**) non-cancer controls. The platelet versus plasma particles in (**a**) shows that The TRAP6-activated platelets form a common group (cluster 3) with some platelet and plasma particles from Twente. Some of the platelet particles from Twente also overlap with the control EVs from Paris (cluster 1). The A23187-activated platelet EVs form a distinct group with minor overlap with the TRAP6-activated platelet EVs (cluster 2). The cancer vs. control shown in (**b**) demonstrates that the cancer controls form a large cluster with the cancer patients forming distinct clusters surrounding it. The individual cancer patients in (**c**) show that patients three and four form a common group, but the remaining patients form distinct groups. The individual controls shown in (**d**) show that all but control 5 form a common group, with control 5 forming a distinct outlier group.

The plot in Fig. 6 shows six clusters, indicating that the model sees varying degrees of similarity and dissimilarity between the particles. A similar plot, using PCA, is shown in Fig. S4. Due to the large number of datapoints and labels, it is difficult to discern them in Fig. 6. For better clarity, a few labels have been selected and plotted in Fig. 7. Some more selections can be found in Fig. S5. Analysis of platelet-derived particles shows that these form three distinct groups, as shown in Fig. 7a. The TRAP6-activated platelets form cluster 3 with the platelet particles from Twente, indicating that the model sees them as similar despite the fact that they were generated at two different labs. Cluster 3 also overlaps with the plasma-derived particles, indicating a similarity between them. The control platelets from Paris form cluster 1, separated from the activated platelets, indicating that the model sees a difference between them. Cluster 1 also overlaps with the platelet particles from Twente. The A23187-activated platelet-derived EVs in cluster 2 has only minor overlap with the TRAP6-activated platelet-derived EVs, indicating that the model sees a difference in the particles depending on both the activation state of the platelets and the method of activation. Furthermore, the model demonstrates that it sees a distinct difference between the cancer patients and controls, as shown by the clustering in Fig. 7b. The cancer patient-derived particles, shown in Fig. 7c, shows that the model sees patients 3 and 4 as similar by them forming a common group while the remaining patients form distinct groups. For the cancer controls, shown in Fig. 7d, it is shown that controls 1–4 form a common group, indicating that the model sees them as similar despite the particles originating from different donors. However, the particles from control 5 form a distinct cluster, indicating that the model sees something different about them that more closely resemble what it sees in cancer patients 3 and 4.

### Classification accuracy

**Figure 8:**
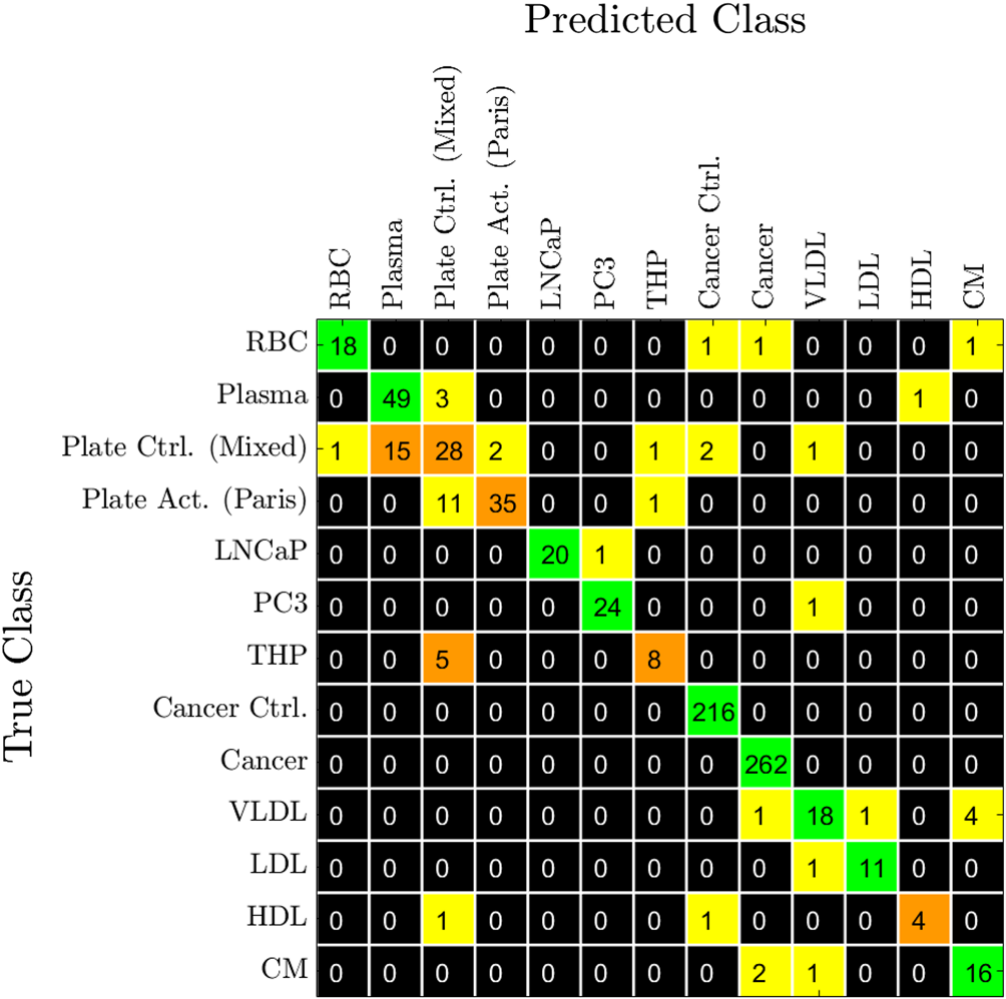
Confusion matrix for the classifier trained on the latent space representations of the spectra. The colors indicate the percentage correct predictions, with yellow for 1$\%$ to 25$\%$ correct, orange for 25$\%$ to 50$\%$ correct, and green for more than 75$\%$ correct prediction. The numbers show the number of analysed spectra for each case.

After the classifier has been trained on the latent representation of the training set produced by the encoder, the test set is passed through the encoder and to the classifier. The classifier is trained to recognize the same origins as shown by the labels in Fig. 6 except for the merging of the platelet particles from Twente and platelet control particles from Paris into one label. The resulting confusion matrix of the classifications are shown in Fig. 8, with supplementary matrices with percentages and for ICA in Figs. S4–S6. As an example from the matrix, it shows that the cancer patients can be perfectly differentiated from non-cancer controls by the model. This agrees with the differentiation shown in Fig. 7b. Note that the classifier observes all 100 dimensions of the data instead of the compressed 2D t-SNE representation shown in Fig. 7b, thus the classifier can differentiate the points in dimensions that cannot be illustrated here. In cases of EVs from bulk blood, the model is also able to differentiate particles from blood plasma and red blood cells with a high degree of accuracy, producing no misclassification between the two. Over a total of 769 spectra in the test set 709 are correctly classified, yielding a true positive rate (sensitivity) of 92.2$\%$ and true negative rate (selectivity) of 92.3$\%$. Note that the highest rate of mutual misclassification is between the control platelet- and plasma-derived particles, which agrees with the differentiation shown in Fig. 7a. The noise robustness of the classifier is also tested by introducing the same clipping to the test data as for the training data, resulting in a sensitivity of 84.7$\%$ and a selectivity of 86.2$\%$ for clipped data. Further degradation of the data by noise or frequency distortions similar to the training case resulted in the classifier maintaining a sensitivity of 81.5$\%$ and a selectivity of 83.8$\%$ or higher. The resulting confusion matrices are shown in Fig. S4.

## Discussion

In this work we have constructed a novel machine learning model that is able to reconstruct the Raman spectra of EVs with a high degree of accuracy and with strong de-noising. The model is shown to be very flexible, as it can reconstruct well data from different origins, with differences in frequency range and in noise level. The extracted features are of high quality, enabling to classify and cluster EVs and lipoproteins based on their cellular origin and activation state, as well as an overall condition (health/disease). This is verified by the classifier, which attains more than 90 $\%$ accuracy in classifying the biological nanoparticles to their correct origins.

In the reconstructions shown in Fig. 5, we see that the autoencoder perceives all of the significant features and reconstructs them well; both the low-frequency slopes and complexes, as well as the high-frequency peaks. From the frequency range reconstructions shown in Fig. 5b we see that the frequency vectors are reconstructed well, despite some degree of error. This error can readily be attributed to the weighting of the learning process, where the intensity vector, containing the actual Raman features, is given more importance in reconstruction. Nevertheless, the adaptive behavior of the reconstructed frequency vector clearly shows that the model actively considers it in its determination of the features in the spectrum and that the model can adapt to data with very different frequency ranges from different labs.

Figures 6 and 7 show that the extracted features form natural clusters that correspond well with the samples that produced the spectra. The most notable of these results is shown in Fig. 7a. For the dataset from Paris, EVs from non-activated control platelets (Plate Ctrl., cluster 1 in Fig. 7a) are well differentiated from the EVs from activated platelets. The model further recognizes the difference between activation with TRAP6 (Plate Act. Trap, cluster 3 in Fig. 7a) or A23187 (Plate Act. A23, cluster 2 in Fig. 7a). For the dataset from Twente, platelet particles overlap with both the controls from Paris and the Paris particles derived from platelets activated with TRAP6. The overlap in the platelet particles from Twente and Paris, despite very different noise levels and frequency ranges, illustrates the achieved strength and flexibility of the model for variations in the measurement method. The separate clusters for EVs from platelets activated with TRAP6 (Trap) and with A23187 (A23), both Paris, further emphasizes the model's ability to differentiate EVs. The proximity of the platelets from Twente to the plasma particles also indicates that the model sees them as similar. This can be due to the preparation of the particles. The EVs analysed in the Paris lab were isolated from washed platelets isolated from fresh blood. In contrast, the Twente platelet particles were prepared from platelets recovered from platelet concentrates^33^, which has been reported to have higher levels of activated platelets. Regarding Fig. 7d, four of the (non-cancer) controls are clustered together, indicating that the model can see them as similar despite originating from different controls. But one control forms a tight, separate cluster, indicating that the model sees a difference relative to controls 1-4. As the medical record and possible medication of the controls is not known, this difference cannot be further investigated.

Previous studies have shown that EVs from different cell lines and primary cells carry different biochemical features, as analyzed by Raman spectroscopy and PCA^19,41^. Such analysis, however, did not generate a clear distinction, as shown in Fig. S4. It also could not blindly associate features extracted from Raman spectra of EVs with their cells of origin. The features extracted from the Raman spectra of EVs by the autoencoder enable classification according to the cellular origin, as well as between cancer patients and subjects who do not have cancer. Such classification opens new frontiers and possible uses of EVs for the diagnosis and prediction of disease. Of particular note is the specific identification of EVs and lipoproteins from activated platelets, which were clearly separated from those isolated from resting platelets and red blood cells. This indicates that the model can see a clear difference between particles from active platelets and resting platelets or non-platelets. Platelet activation and subsequent release of EVs is thought to play a role in various thrombotic conditions, such as venous thromboembolism (VTE)^31^. Therefore, it would be of interest to investigate whether our autoencoder and classifier can detect elevated levels of EVs from activated platelets in the plasma of VTE patients and whether this could facilitate future prediction or diagnoses of the disease. The exclusivity of the features of EVs/lipoproteins from platelets was further demonstrated by the model grouping similar nanoparticles from platelets generated in two independent labs, each using different instruments and operators. Our results also warrant further investigation in the field of cancer, where patients exhibit a distinct EV profile.

The quality of the information extracted from the spectra by the model is demonstrated by the achieved accuracy of the classifier, as shown in Fig. 7. By taking the extracted features given by the latent space of the autoencoder, the classifier achieves both a sensitivity and selectivity of over 90$\%$ across the test set. Most notable is the fact that there is no misclassification between the cancer- and control-derived particles, meaning that the model is capable of detecting prostate cancer with perfect sensitivity and selectivity. This indicates that the model perceives a significant difference between the EVs from cancer compared to non-cancer. The classifier also gives an 82.3$\%$ accuracy in classifying platelet-derived EVs as activated or resting, with a sensitivity of 76.1$\%$ and selectivity of 93.3$\%$ for detection of activated platelets. The lower sensitivity of this test indicates that the model sees a relatively small difference between the activated and un-activated platelet-derived EVs, which can be expected given their intrinsic similarity as they have a common origin. This is also illustrated in Fig. 7a by the partial overlap between the platelet particles from Twente and non-activated platelets from Paris, as discussed above. There is also a significant overlap between the platelets from Twente and the plasma-derived particles, resulting in a sensitivity of only 65.1$\%$ for detecting control platelets from plasma.

## Conclusion

We have demonstrated that a self-supervised deep learning model with a carefully taylored structure and loss function can learn to reconstruct a wide variety of Raman spectra with a highly variable signal-to-noise ratio and with a variable frequency range. The ability to consider spectra from separate measurement systems, while maintaining high reconstruction accuracy, underscores the versatility of the model. The de-noising performance of the model is also shown to be promising, leaving a residual difference between the spectra and reconstructions that follows a Gaussian distribution, indicating that the residual is largely random noise that is filtered out by the model. The model is also shown to be capable of extracting valuable, biochemically significant information from the spectra, which allows it to perform label-free clustering of particles by their similarity despite the significant differences in the measurement methods.

It is shown that the model can recognize EVs from resting platelets and activated platelets as two distinct phenomena, and that it can recognize particles as belonging to either of these two phenomena regardless if they come from the lab in Paris or in Twente. It is also shown capable of recognizing that EVs from platelets activated by Calcium Ionophore (A23187) and platelets activated by Thrombin (TRAP6) are distinct. This demonstrates both the viability of using Raman spectroscopy as a means of detecting platelet activation and the viability of using the demonstrated model to reliably extract valuable information from those spectra. For EVs derived from prostate cancer patients, individual patients are mostly separated, while the EVs from controls are mostly clustered together. The reason for this cannot be further investigated without more data and knowing the medical conditions of the patients and the controls. The model readily differentiates the cancer patients from the non-cancer controls, with a perfect sensitivity and selectivity, demonstrating the efficient and promising combination of Raman spectroscopy and machine learning for this task. In cases of bulk blood, the model is also able to differentiate blood plasma and red blood cells with a high degree of accuracy, producing no misclassification between the two.

### Supplementary Information

Supplementary Information.

## Data Availability

The datasets generated and analysed during the current study are available from the corresponding author on reasonable request.
