# Supplementary material for: Identification of extracellular vesicles from their Raman spectra via self-supervised learning
Source: Sci Rep. 2024 Mar 21;14:6791. doi: 10.1038/s41598-024-56788-7 (PMC10957939; doi:10.1038/s41598-024-56788-7)
Supplement: Supplementary file 1 — Supplementary Information. [file 41598_2024_56788_MOESM1_ESM.pdf]

SUPPLEMENTARY INFORMATION

Identification of extracellular vesicles from their Raman spectra via self-supervised learning

Mathias N. Jensen[1], Eduarda M. Guerreiro[2], Agustin Enciso-Martinez[3,4,5], Sergei G. Kruglik[6], Cees Otto[7], Omri Snir[2,8], Benjamin Ricaud[1], and Olav Gaute Hellesø[1,*]

[1]Department of Physics and Technology, UiT The Arctic University of Norway, Tromsø, Norway.
[2]Thrombosis Research Group (TREC), Department of Clinical Medicine, UiT The Arctic University of Norway, Tromsø, Norway.
[3]Oncode Institute and Ten Dijke/Chemical Signaling Laboratory, Department of Cell and Chemical Biology, Leiden University Medical Center, Leiden, The Netherlands
[4]Amsterdam Vesicle Center, Department of Biomedical Engineering and Physics, Amsterdam University Medical Centers, Amsterdam, The Netherlands
[5]Laboratory of Experimental Clinical Chemistry, Department of Clinical Chemistry, Amsterdam University Medical Centers, Amsterdam, The Netherlands
[6]CNRS, Institut de Biologie Paris-Seine, Laboratoire Jean Perrin, Sorbonne University, Paris, France
[7]Department of Medical Cell BioPhysics, TechMed Centre, University of Twente, Enschede, The Netherlands
[8]Department of Medical Biology, UiT The Arctic University of Norway, Tromsø, Norway
[*]olav.gaute.helleso@uit.no

ABSTRACT

Extracellular vesicles (EVs) released from cells attract interest for their possible role in health and diseases. The detection and characterization of EVs is challenging due to the lack of specialized methodologies. Raman spectroscopy, however, has been suggested as a novel approach for biochemical analysis of EVs. To extract information from the spectra, a novel deep learning architecture is explored as a versatile variant of autoencoders. The proposed architecture considers the frequency range separately from the intensity of the spectra. This enables the model to adapt to the frequency range, rather than requiring that all spectra be pre-processed to the same frequency range as it was trained on. It is demonstrated that the proposed architecture accepts Raman spectra of EVs and lipoproteins from 13 biological sources and from two laboratories. High reconstruction accuracy is maintained despite large variances in frequency range and noise level. It is also shown that the architecture is able to cluster the biological nanoparticles by their Raman spectra and differentiate them by their origin without pre-processing of the spectra or supervision during learning. The model performs label-free differentiation, including separating EVs from activated vs. non-activated blood platelets and EVs/lipoproteins from prostate cancer patients vs. non-cancer controls. The differentiation is evaluated by creating a neural network classifier that observes the features extracted by the model to classify the spectra according to their sample origin. The classification reveals a test sensitivity of 92.2% and selectivity of 92.3% over 769 measurements from two labs that have different measurement configurations.

Sample preparation

For 'Paris' dataset, sample prepared at UiT The Arctic University of Norway and analysed at Sorbonne University

Collection of clinical samples has been approved by the regional ethical committee for Medical and Health Research Ethics (REK200825). All participants were above the age of 18, did not suffer from illness, or use medication; all gave a written informed consent. Blood was drawn by venipuncture of the antecubital vein using a 21-gauge needle and minimal stasis. Blood was collected into Vacuette 6 ml Z tubes with no additives (Greiner Bio-One, Kremsmünster, Austria); the first tube was discarded. Acidic citrate dextrose buffer (ACD, 39 mM citric acid, 75 mM sodium citrate, 135 mM [D]-glucose, pH 4.5) and 2,8 mM Prostaglandin E1 (PGE1, MedChemExpress, Monmouth Junction, NJ, USA) were added rapidly to the blood to prevent blood coagulation and platelet activation, respectively. In addition, 3 ml of blood were drawn into K2EDTA Vacuette tubes (Greiner Bio-One, Kremsmünster, Austria) for cell count and analysis using an ABX MicrosES60 (ABX Diagnostics, Montpellier, France). Following addition of ACD and PGE1, blood was centrifuged at 140 xg for 15 min with no breaks (room temperature) using a Megafuge 1.0 (Heraeus Sepatech) centrifuge equipped with a swing bucket rotor BS4402/A to generate

platelet rich plasma (PRP).

Platelet pellets were recovered from PRP following centrifugation at 900 xg for 15 min at room temperature, washed twice with HEPES-NaCl buffer (10 mM HEPES, 0.85% NaCl, pH 7.4) and 2.8 mM PGE1, and resuspended in Tyrode-HEPES buffer (10 mM HEPES, 0.85% NaCl, 1 mM MgCl2, 2 mM CaCl2, 3 mM KCl, pH 7.4). Platelets (250x106 in 1 ml Tyrode-HEPES buffer) were stimulated with 100 µM thrombin receptor activator peptide 6 (TRAP-6, MedChemExpress, Monmouth Junction, NJ, USA) or 2 µM calcium ionophore A23187 (Sigma-Aldrich, USA) and incubated for 15 min at 37°C. Saline was added for time-matched unstimulated control platelets. Following 15 min, EDTA was added to platelet suspensions (activated and time-matched control) at a final concentration of 10 mM to stop platelet activation and platelets were sedimented at 2,500 xg for 10 min at room temperature. Supernatant was transferred to a new tube before proceeding with isolation of platelet-Evs, and the platelet pellets were resuspended in 1% paraformaldehyde (PFA) in PBS for assessment of platelet activation by flow cytometry. Platelet derived (micro)particles, hereafter referred to as platelet-Evs, were isolated from supernatant by centrifugation at 20,000 xg for 30 min at 4°C, using a 5810R Eppendorf centrifuge with a fixed angle rotor FA-45-30-11. EV pellets were resuspended in 1/10 of their initial volume (i.e., 10x concentrated) in a buffer suitable for the respective downstream analysis.

For 'Twente' dataset, sample prepared and analysed at University of Twente

Plasma from healthy donors and cancer patients

Blood was obtained from non-fasting healthy donors and mCRPC patients (both N=5) after written informed consent in accordance with the Helsinki Declaration and approved by the medical-ethical assessment committee of the Academic Medical Center, University of Amsterdam (NL 64623.018.18). Refer to Table S1 for donors clinical data. Whole blood was collected from each donor using a 21G needle, and the first vacutainer was discarded. Next, three citrate vacutainers of 2.7 mL (BD Biosciences, San Jose, CA) were collected and mixed gently by inversion. The vacutainers were centrifuged at 2500 g for 15 minutes at 20 °C without brake (Rotina 380R, Hettich, Tuttlingen, Germany). Plasma was collected up to 0.5 cm above the pellet, pooled and centrifuged in a conical base tube (10 mL; Sarstedt, Nimbrecht, Germany) at 2500 g for 15 minutes at 20 °C. The supernatant was deposited in aliquots of 75 µL (Sarstedt), which were snap frozen in liquid N2 and stored at 80 °C until use. Samples were thawed in a water bath at 37 °C immediately before use.

Lipoprotein particles (LPs) Human high density lipoprotein (HDL), low density lipoprotein (LDL), very low density lipoprotein (VLDL) and chylomicrons (CM) were acquired from Sigma-Aldrich Chemie N. V. (The Netherlands). HDL (Cat. No.: L8039), LDL (Cat. No.: 437644), VLDL (Cat. No.: 437647) and CM (Cat. No.: SRP6304) had a purity of $\geq$ 95% by electrophoresis, as specified by the provider.

LNCaP-derived EVs Cells from the prostate cancer cell line LNCaP (ATCC, CRL-1740, USA) were cultured at 37°C and 5% CO2 in RPMI-1640 with L-glutamine medium (Lonza, Cat. No.: 12-702F) supplemented with 10% (v/v) fetal bovine serum (FBS), 10 units/mL penicillin and 10 mg/mL streptomycin. Cells were seeded at a density of 10,000 cells/cm2 as recommended by ATCC and medium was refreshed every second day. At 80-90% confluence, cells were washed three times with phosphate buffer solution (PBS) and cultured in FBS-free RPMI-1640 with L-glutamine medium (Lonza, Cat. No.: 12-702F) supplemented with 1 unit/mL penicillin and 1µg/mL streptomycin. After 2-3 days of culture, cell supernatant was collected in a 15 mL tube (Cellstar® tubes, Grenier Bio-one BV, Alphen a/d Rijn, The Netherlands) and centrifuged at 500 g at room temperature for 10 minutes (centrifuge 5804, Eppendorf, Hamburg, Germany). Next, the supernatant containing LNCaP-derived EVs was collected and stored in aliquots (Greiner Bio-one) at -80 °C until use. Samples were thawed in a water bath at 37 °C immediately before use. LNCaP-derived EVs are referred to as LNCaP EVs throughout the text.

Red blood cell (RBC) - derived EVs RBC-derived EVs were obtained from RBC concentrate (150 mL, Sanquin Bloodbank, Amsterdam, The Netherlands) and diluted 1:1 with filtered PBS. Samples were centrifuged three times at 1560 g for 20 minutes at 20 °C (Rotina 46RS centrifuge, Hettich, Tuttlingen, Germany). The supernatant containing EVs was pooled and distributed in aliquots of 50 µL, which were snap frozen in liquid N2 for 15 minutes and stored in aliquots (Sarstedt) at -80 °C until use. Samples were thawed in a water bath at 37 °C before use. RBC-derived EVs are referred to as RBC EVs throughout the text.

For other particles used from Twente, see the methods-section of Martinez *et al.*[1]

Table S1. Clinical data on the cancer patients afflicted with Metastasized castration-resistant cancer (mCRPC) and control donors.

Subject	Date of diagnosis	Date of CRPC diagnosis	Treatment	Age	PSA*	LDH	ALP	tChol	HDL	LDL	TGL
Pt 1	05/2017	10/2018	Abiterone Prednisone	66	0.6	202.9	1.6	3.89	1.27	2.13	1.08
Pt 2	2006	04/2018	Enzalutamide	86	3.4	165.4	1.4	5.44	1.57	3.24	1.41
Pt 3	2000	04/2018	Abiterone Prednisone	75	1.2	175.6	5.0	3.14	1.37	1.39	0.86
Pt 4	NA	NA	NA	77	17.6	165.3	3.3	5.45	2.22	2.82	0.92
Pt 5	NA	NA	NA	82	10.4	240.3	6.4	5.75	1.36	3.59	1.78
HD min	NA	NA	NA	19	NA	125.8	1.2	3.45	0.93	1.81	0.42
HD max	NA	NA	NA	40	NA	270.3	3.2	4.38	1.45	2.47	1.93

Pt: patient, HD: healthy donor (N=5), PSA: prostate specific antigen *last determined PSA level before inclusion
LDH: Lactate dyhydrogenase (U/L 37C), ALP: Alkaline phospatase (U/L 37C), tChol: total cholesterol (mmol/L)
HDL: high density lipoproteins (mmol/L), LDL: low density lipoproteins cholesterol (mmol/L), TGL: Triglyceride (mmol/L).

Dataset

The available dataset consists of a total of 2 684 spectra of extracellular vesicles and lipoproteins, distributed by their origin as listed in Table S2. Due to the data originating from several works on the subject, the number of spectra from different origins varies greatly. Furthermore, due to the measurements originating from two different measurement systems, the wavenumber range of the acquisitions also varies significantly.

Table S2. Overview of samples in the data set with number of spectra, distribution and ranges. The heterogeneity in frequency range is clear, with some having a range of approximately 3300 cm^{-1} and some with a range of approximately 1700 cm^{-1}. There is also a large variability in the number of spectra for each of the origins, with 898 spectra of cancer patient derived particles and only 19 from high density lipoproteins.

Origin/Properties	Subspecies	N_{spect}	$\tilde{\nu}$ min (cm^{-1})	$\tilde{\nu}$ max (cm^{-1})
Plasma		153	301	3672
Cancer ctrl.		745	300	3652
Cancer		898	300	3652
RBC		56	300	3668
Platelet	Ctrl. (Paris)	82	307	2036
	Act. A23 (Paris)	77	308	2035
	Act. Trap (Paris)	81	307	2035
	Mixed (Twente)	184	300	3671
THP		41	315	2041
LNCaP		74	300	3673
PC3		94	300	3668
CM		64	300	3648
HDL		19	300	3652
LDL		47	302	3652
VLDL		69	300	3648

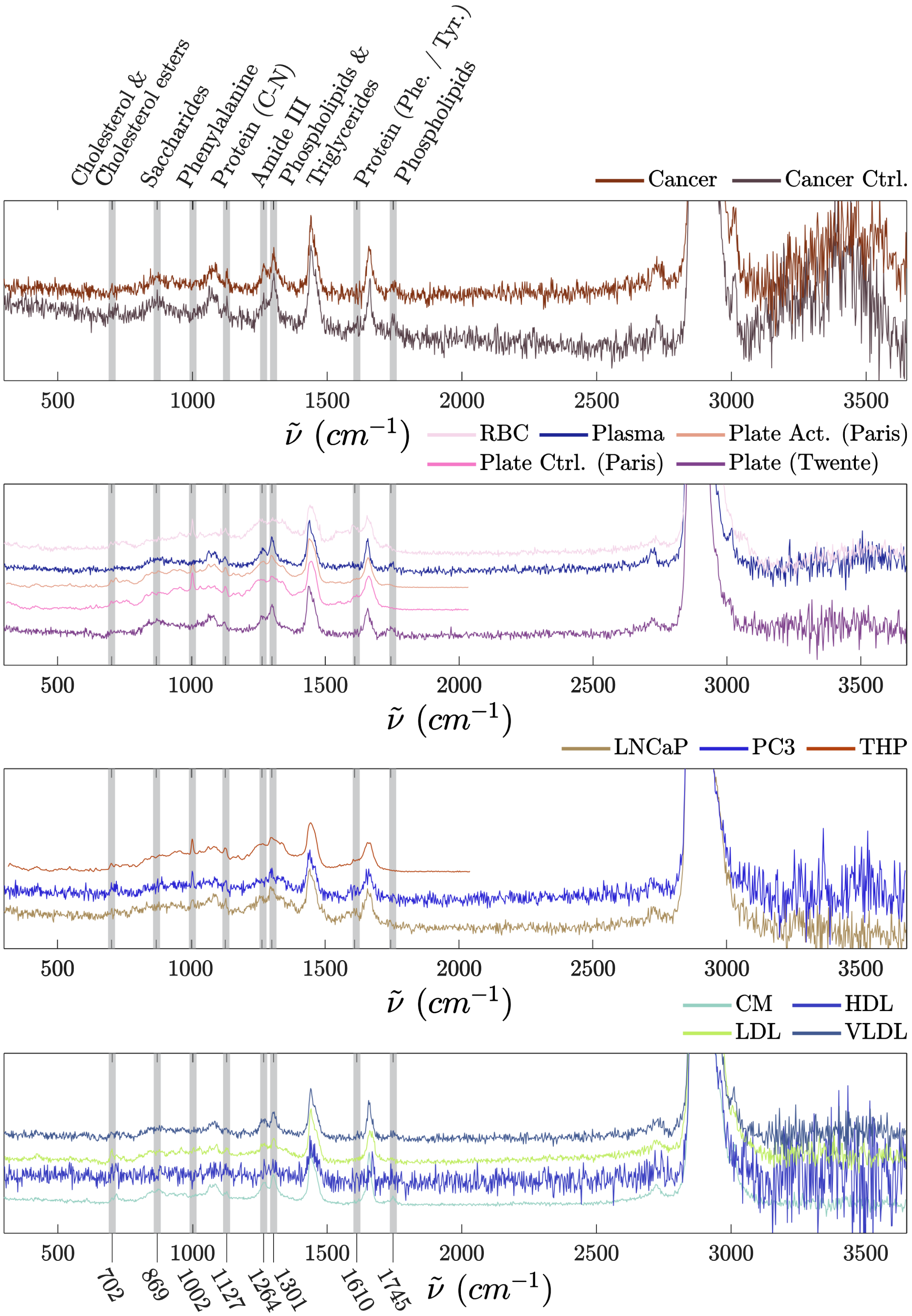

Figure S1. Averaged spectra of EVs/lipoproteins of each biological origin with features of interest marked. The magnitude of the spectra is normalized across the dataset such that they can be compared directly. All spectra are displayed in their full length and resolution without spectral filtering or smoothing.

The mean Raman spectra of the classes listed in Table S2 are shown in fig. S1, with the A23- and Trap-activated platelet derived EVs merged into the class "Plate Act. (Paris)" in fig. S1. Eight spectral bands are selected to illustrate the difference between the spectra of the classes:

- Cholesterol and Cholesterol esters[2] @ 702cm^{-1}: Cholesterol has been detected in exosomes and is theorized to have a relevance in cancer growth[3].
- Saccharides[4] @ 809cm^{-1}: Saccharides, and associated molecules (glycans), have been shown to play a role in the uptake of EVs[5].
- Phenylalanine[6] @ 1002cm^{-1}: Phenylalanine is a common amino acid, making it a useful indicator for proteins.
- Protein (C-N)[7] @ 1127cm^{-1}: C-N streching in protein is used as a marker for protein content.
- Amide III[8] @ 1264cm^{-1}: Amides, among other things, serve to bind amino acids to form proteins and peptides.
- Phospholipids and Triglycerides[9] @ 1301cm^{-1}: Triglycerides (fatty acids) are a major group of lipids and Phospholipids are common in bilayered lipids.
- Protein (phenylalanine and tyrosine)[10] @ 1610cm^{-1}: Proteins are common in EVs and their constituent amino acids, such as phenylalanine and tyrosine, are indicators of proteins.
- Phospholipids[10] @ 1745cm^{-1}: Phospholipids are common in the outer membrane of bilayered lipids, such as EVs.

As is shown in fig. S1, the listed features of interest appear in the spectra and their magnitudes vary by the class of the particles. The spectra are thus evaluated in these bands to evaluate the content of the listed chemicals.
The spectra are evaluated at the bands illustrated in fig. S1, resulting in the magnitudes illustrated in fig. S2. To mitigate bias in the measurements, the spectra are offset to have a base level of zero and a root mean square value of one on a per-spectrum basis. The bars in fig. S1 illustrate the mean magnitudes in each class for each band. The standard deviation of the magnitudes in the spectra of each class is illustrated by brackets surrounding the bar.
While the mean values show a difference between the classes, the difference is small relative to the intra-class standard deviation shown by the brackets. Thus, making accurate classification using these metrics is challenging.

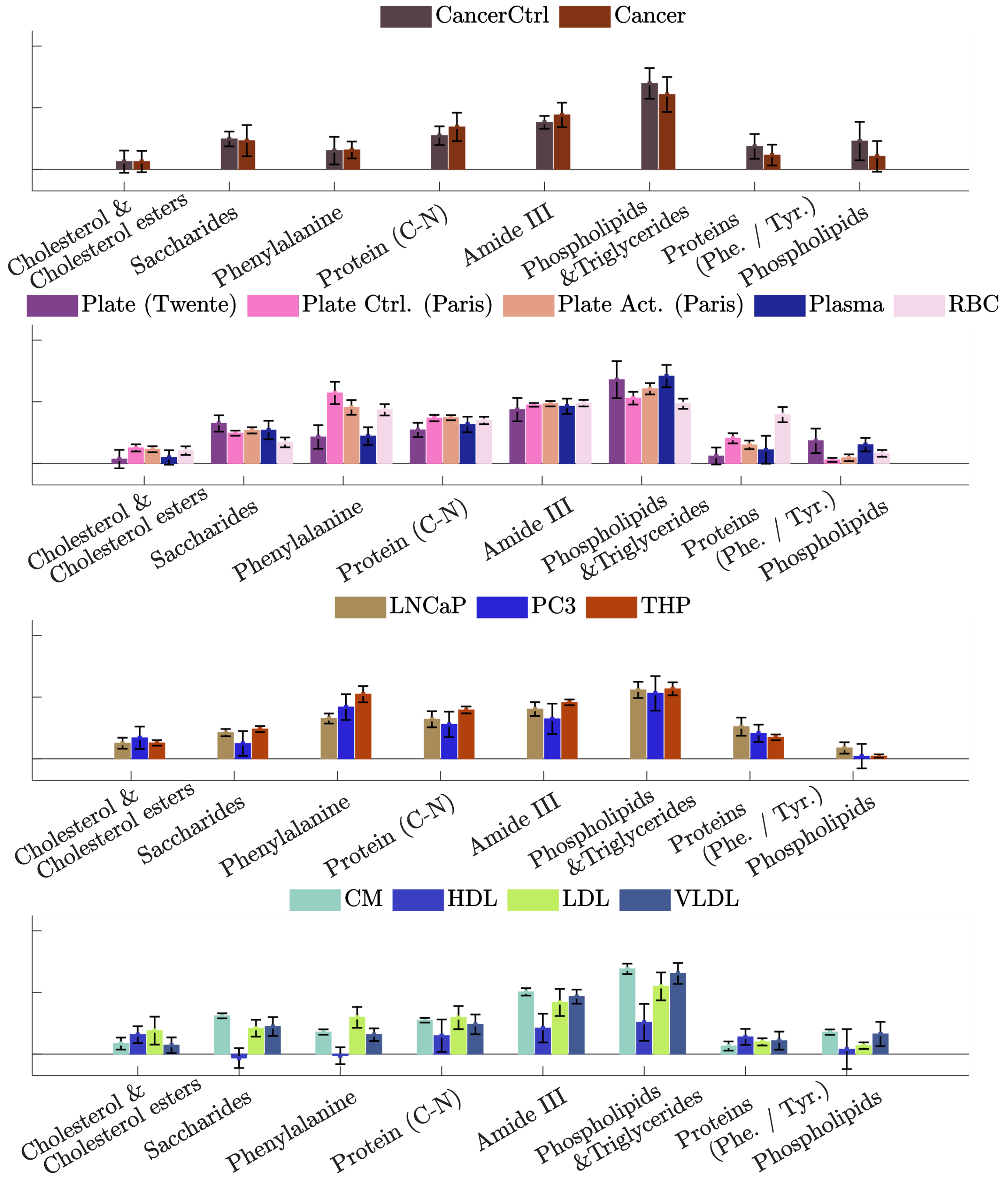

Figure S2. Magnitude and standard deviation of features of interest in the spectra. Bars indicate the mean magnitude of each feature in each class and brackets indicate one standard deviation surrounding the mean values.

Architecture

As described in the main work, the model is built using a modular architecture whose structure is determined by a set of prescribed hyperparameters. Table S3 lists the structure generated by the hyperparameters used to build the model, and the number of learned parameters the elements in the model contain. As described in Table S3, the encoder is significantly larger than the decoder (2 786 419 vs. 917 150 parameters). This is due to the bulk of the models de-noising is done by the encoder, while the decoder presumes that the data in the latent space is de-noised.

Table S3. The final architecture of the encoder and decoder with the total number of parameters.

Element	Block	Layers	Filters	Width	Parameters
Encoder	Conv. Block E1	6	32	5	30 912
	Conv. Block E2	6	64	3	74 112
	Conv. Block E3	4	128	3	197 120
	Feed forward EFreq.	3	64	1	8 704
	Feed forward E	4	512	1	2 475 571
	Latent space	1	1	110	0
Decoder	Feed Forward DFreq.1	3	64	1	9 024
	Feed forward D	4	512	1	872 448
	Feed Forward DFreq.2	1	5	1	30
	Conv. Block D1	2	64	3	12 992
	Conv. Block D2	3	32	3	22 656
Classifier	Feed forward C	5	128	1	78 976

Supplementary results

In addition to the results shown in the main work, intermediate results and reference results were also produced, these are presented here for reference.

Reconstruction

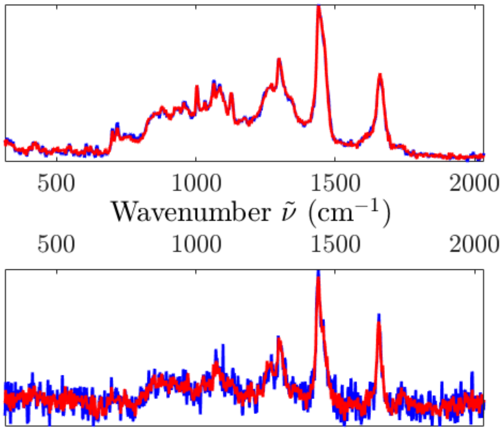

(a) Reconstruction by principal component analysis.

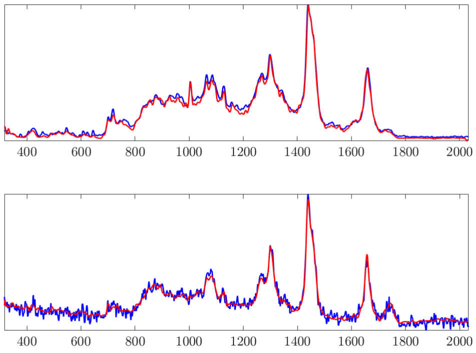

(b) Reconstruction by a simple autoencoder model without skip or explicit frequency range consideration.

Figure S3. Reconstruction performance of comparative methods using examples from the Paris dataset (top) and Twente dataset (bottom). The PCA reconstruction shown in a) is limited to 100 components, same as the autoencoder, and results in the reconstruction being highly accurate, but also preserves a significant amount of noise. For the autoencoder reconstruction shown in b) it is shown that the accuracy is high, and the process eliminates a significant amount of noise but the lack of adaptivity to frequency removes a significant portion of the spectra, as shown by the absence of noise in the reconstruction (red line).

As shown in fig.S3a, using the first 100 components produced by PCA allows for reconstruction of the spectrum with a high degree of accuracy. It is notable that the PCA reconstruction of the low SNR data also reproduces a significant amount of noise, resulting in poor de-noising performance. Comparison with the reconstruction by the base version of the autoencoder (no skip connections or explicit frequency consideration), as shown in fig. S3b, shows a more significant reduction in noise than the PCA reconstruction. This illustrates that even without the more advanced features of the model, it still demonstrates good de-noising performance.

Clustering

For reference, the data used in the main work is processed by PCA to yield the scatter plot shown in fig. S4 using the same methodology as was used to create the scatter plots in figs. 6 and 7 in the main work. Unlike in the main work, the scatter plot of the PCA does not produce the same degree of clustering as the autoencoder demonstrates. In the PCA projetion, the classes are spread out in the space such that they form mixed, thin groups spread out along a circular perimeter.

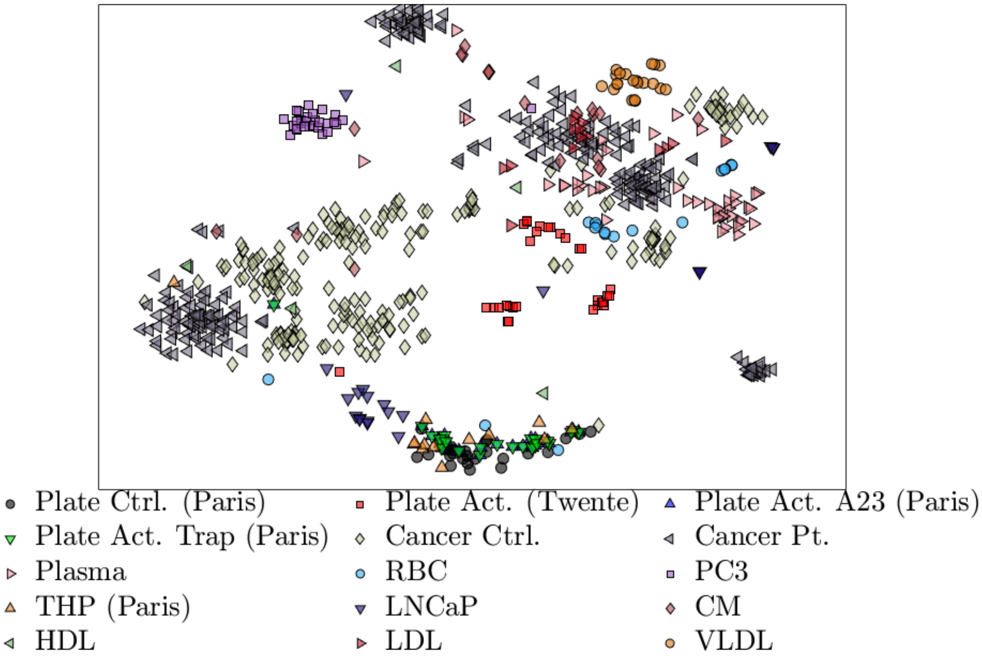

Figure S4. t-SNE projection of PCA components for test data. The particle origins cluster chaotically, forming several smaller clusters, notably the smeared cluster at the center bottom which reflect spectra from Paris. The clusters are highly intermixed, similar to the autoencoder results, but the PCA results are more spread out. See especially the particles from PC3,LNCaP, and THP-1 which are differentiated by the PCA despite their intrinsic similarity and unlike the autoencoder, which recognizes them as similar.

In addition to the PCA scatter plot, additional scatter plots produced by the autoencoder are included, as shown in fig. S5. The plots are subsets from the plot shown in fig. 6 of the main work in the same manner as fig. 7 of the main work. Fig. S5a illustrates that the particles derived from THP and PC3 overlap greatly and form a separate supercluster from that of the sparser cluster of the LNCaP-derived particles. The plot of lipoproteins shown in fig. S5b shows that the chylomicrons overlaps with some of the high-density lipoproteins to form a common group with a subset of the very low density lipoproteins. The majority of the very low density and the high-density lipoproteins form two distinct groups separate from the mixed group. The plot of the blood elements shown in fig. S5c shows good separation between the red blood cells and the plasma-derived particles.

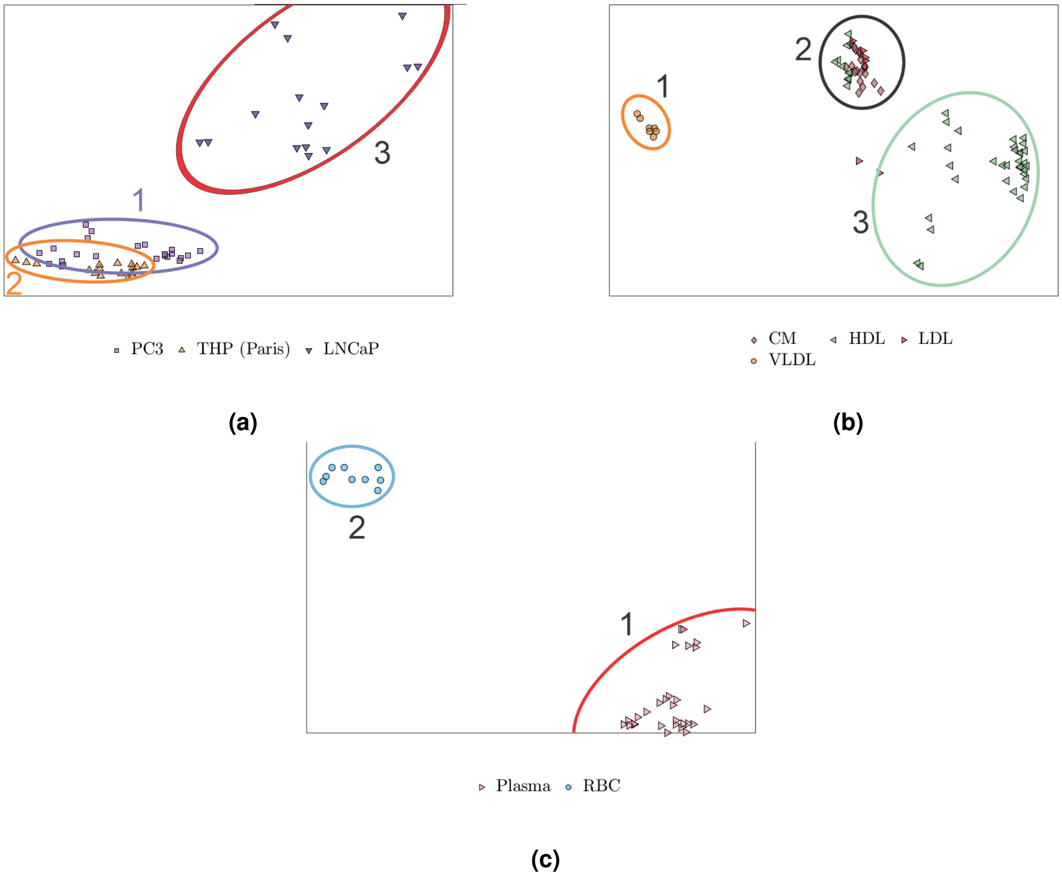

Figure S5. t-SNE projection of the latent space for the autoencoder and for various selections of EVs and nanoparticles. The cultivated cells shown in a) exibit good separation of the LNCaP cells (cluster 3) from the remaining, but shows significant overlap between the PC3 and THP cells (clusters 1 and 2). The lipoproteins shown in b) demonstrate good separation of the HDL (cluster 3) and VLDL (cluster 1) lipoproteins, but some of the VLDL particles are mixed with the CM and HDL (cluster 2). The blood elements shown in c) exhibit excellent separation between the plasma and RBC particles, with no overlap.

Classification

In addition to the confusion matrix shown in fig. 8 of the main work, additional tests were performed using altered data to evaluate the robustness of the classification. The confusion matrix in fig. S6a is the same as fig. 8 in the main work, but showing the classification as percentage instead of number of spectra. Fig. S6b illustrates the classification accuracy with the same test data, but with artificial range clipping as was done during training of the autoencoder and described in the main work under "Training scheme". High accuracy is maintained with range clipping, but a higher number of slight misclassifications occur, resulting in a higher number of spectra of each class being misclassified than was the case for the unaltered testing data. The final confusion matrix shown in fig. S6c is generated from the test data with clipping, noise, and distortion, as was done during training. This matrix shows further degradation of the accuracy and a higher number of misclassifications, but the overall accuracy remains relatively high.

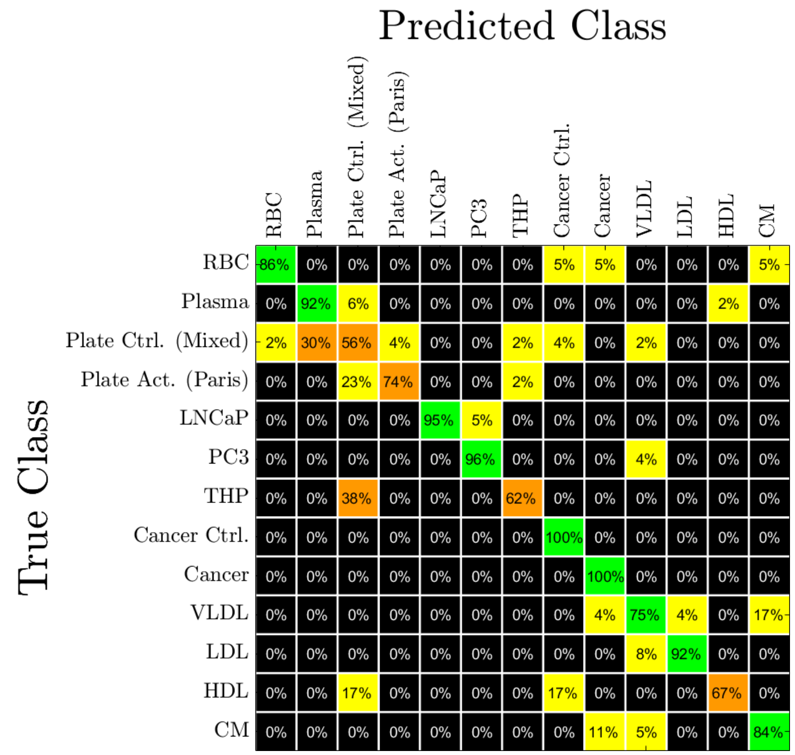

(a) Confusion matrix for unaltered test data.

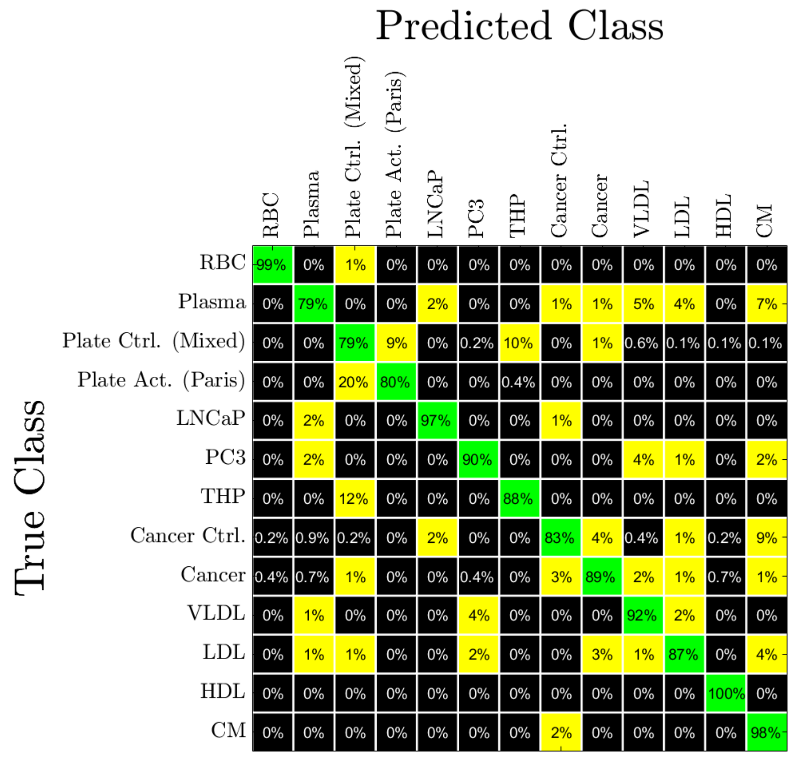

(b) Confusion matrix for randomly clipped test data

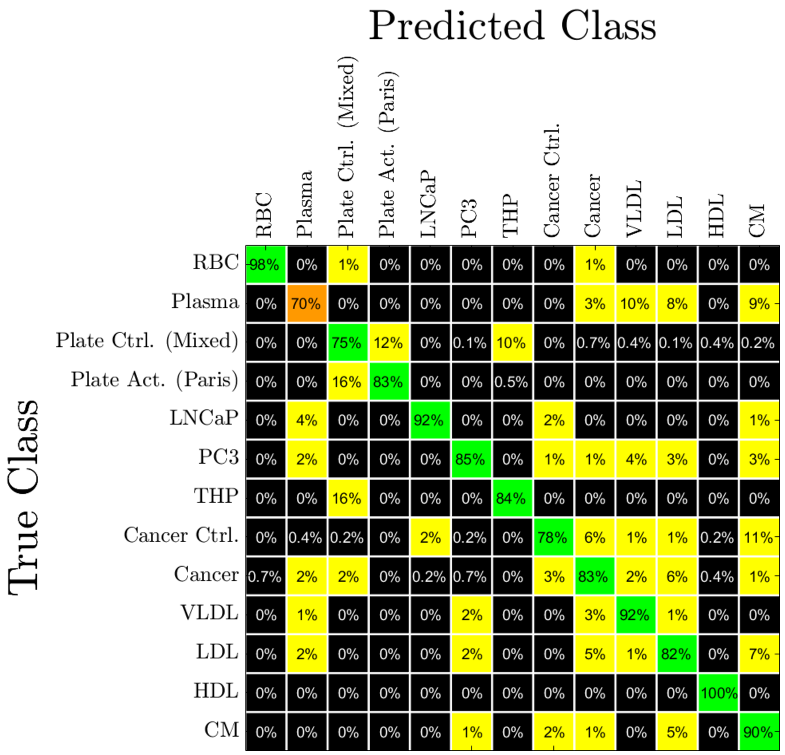

(c) Confusion matrix for test set with clipped and noisy test data.

Figure S6. Confusion matrices for noise augmented test sets. The percentages are given by the number of true labels, e.g. 66% RBC indicates that 66% of the true RBC spectra are classified as RBC by the model. The confusion matrix in a) shows the results of classification on the source data of the test set, without any artificial distortion or noise. The confusion matrix in b) shows the results of classification on the test set augmented with random clipping of the spectra, as was done to the training set during learning. The results show a general reduction in accuracy due to increased misclassification, indicated by yellow, but remains generally good, indicated by the green on the diagonal. The confusion matrix in c) shows the result of classification on the test set augmented with random clipping, intensity noise, and frequency distortion in the same manner as the training set during learning. The accuracy is further reduced, indicated by the orange and yellow, due to increased misclassification. The colors indicates the percentage correct predictions, with yellow for 1% to 25% correct, orange for 25% to 50% correct, and green for more than 75% correct prediction.

References

1. Enciso-Martinez, A. *et al.* Label-free identification and chemical characterisation of single extracellular vesicles and lipoproteins by synchronous rayleigh and raman scattering. *J. Extracell. Vesicles* **9**, 1730134, DOI: 10.1080/20013078.2020.1730134 (2020).
2. Smith, Z. J. *et al.* Single exosome study reveals subpopulations distributed among cell lines with variability related to membrane content. *J. Extracell. Vesicles* **4**, 28533, DOI: 10.3402/jev.v4.28533 (2015). PMID: 26649679, https://doi.org/10.3402/jev.v4.28533.
3. Record, M., Poirot, M. & Silvente-Poirot, S. Emerging concepts on the role of exosomes in lipid metabolic diseases. *Biochimie* **96**, 67–74, DOI: https://doi.org/10.1016/j.biochi.2013.06.016 (2014). Lipids in Metabolic Diseases.
4. Krafft, C., Neudert, L., Simat, T. & Salzer, R. Near infrared raman spectra of human brain lipids. *Spectrochimica Acta Part A: Mol. Biomol. Spectrosc.* **61**, 1529–1535, DOI: https://doi.org/10.1016/j.saa.2004.11.017 (2005). Honour Issue - Jim Durig.
5. Williams, C. *et al.* Glycosylation of extracellular vesicles: current knowledge, tools and clinical perspectives. *J. Extracell. Vesicles* **7**, 1442985, DOI: 10.1080/20013078.2018.1442985 (2018). PMID: 29535851, https://doi.org/10.1080/20013078.2018.1442985.
6. Hernández, B., Pflüger, F., Kruglik, S. G. & Ghomi, M. Characteristic raman lines of phenylalanine analyzed by a multiconformational approach. *J. Raman Spectrosc.* **44**, 827–833, DOI: https://doi.org/10.1002/jrs.4290 (2013). https://analyticalsciencejournals.onlinelibrary.wiley.com/doi/pdf/10.1002/jrs.4290.
7. Zhu, S., Cui, X., Xu, W., Chen, S. & Qian, W. Weighted spectral reconstruction method for discrimination of bacterial species with low signal-to-noise ratio raman measurements. *RSC Adv.* **9**, 9500–9508, DOI: 10.1039/C9RA00327D (2019).
8. Schweitzer-Stenner, R. *et al.* Structure analysis of dipeptides in water by exploring and utilizing the structural sensitivity of amide iii by polarized visible raman, ftir- spectroscopy and dft based normal coordinate analysis. *The J. Phys. Chem. B* **106**, 4294–4304 (2002).
9. Oleszko, A. *et al.* Comparison of ftir-atr and raman spectroscopy in determination of vldl triglycerides in blood serum with pls regression. *Spectrochimica Acta Part A: Mol. Biomol. Spectrosc.* **183**, 239–246, DOI: https://doi.org/10.1016/j.saa.2017.04.020 (2017).
10. Zanyar Movasaghi, S. R. & Rehman, I. U. Raman spectroscopy of biological tissues. *Appl. Spectrosc. Rev.* **42**, 493–541, DOI: 10.1080/05704920701551530 (2007). https://doi.org/10.1080/05704920701551530.
